# Virtual Residency Interviews: Optimization for Applicants

**DOI:** 10.7759/cureus.11170

**Published:** 2020-10-26

**Authors:** Benjamin A Sarac, Kevin Calamari, Jeffrey Janis

**Affiliations:** 1 Plastic and Reconstructive Surgery, The Ohio State University College of Medicine, Columbus, USA; 2 Emergency Medicine, The Ohio State University College of Medicine, Columbus, USA; 3 Plastic and Reconstructive Surgery, The Ohio State University Wexner Medical Center, Columbus, USA

**Keywords:** virtual, interview, covid-19, residency, application

## Abstract

Social distancing guidelines during the coronavirus disease 2019 (COVID-19) pandemic have created substantial changes in undergraduate medical education in the United States. Specifically, the Coalition for Physician Accountability recommended that all programs transition to online interviews and visits for the 2020-21 application cycle. Current literature lacks concrete recommendations with visual examples for how interviewees should best prepare their interview rooms. The authors present cost-conscious recommendations addressing three main areas: the interview room background/environment, audiovisual quality, and virtual interview etiquette, while providing two before and after intervention photographs. Through optimization of these three domains, applicants can present the best versions of themselves during virtual residency interviews.

## Introduction

Social distancing guidelines as a result of the severe acute respiratory syndrome coronavirus 2 (SARS-CoV-2) (coronavirus disease 2019 (COVID-19)) pandemic have created substantial curricular changes in undergraduate medical education in the United States [1]. Despite curricular changes, the effects of the pandemic on the 2020-21 residency application cycle are unknown [2,3]. However, on May 11, 2020, the Coalition for Physician Accountability recommended that all programs transition to online interviews and visits for the current application cycle [4]. This change places an additional burden on prospective residents, as virtual interviews require applicants to set up their own interview rooms. Unfortunately, current literature lacks concrete recommendations with visual examples for interviewees [5].

## Technical report

**Figure 1:**
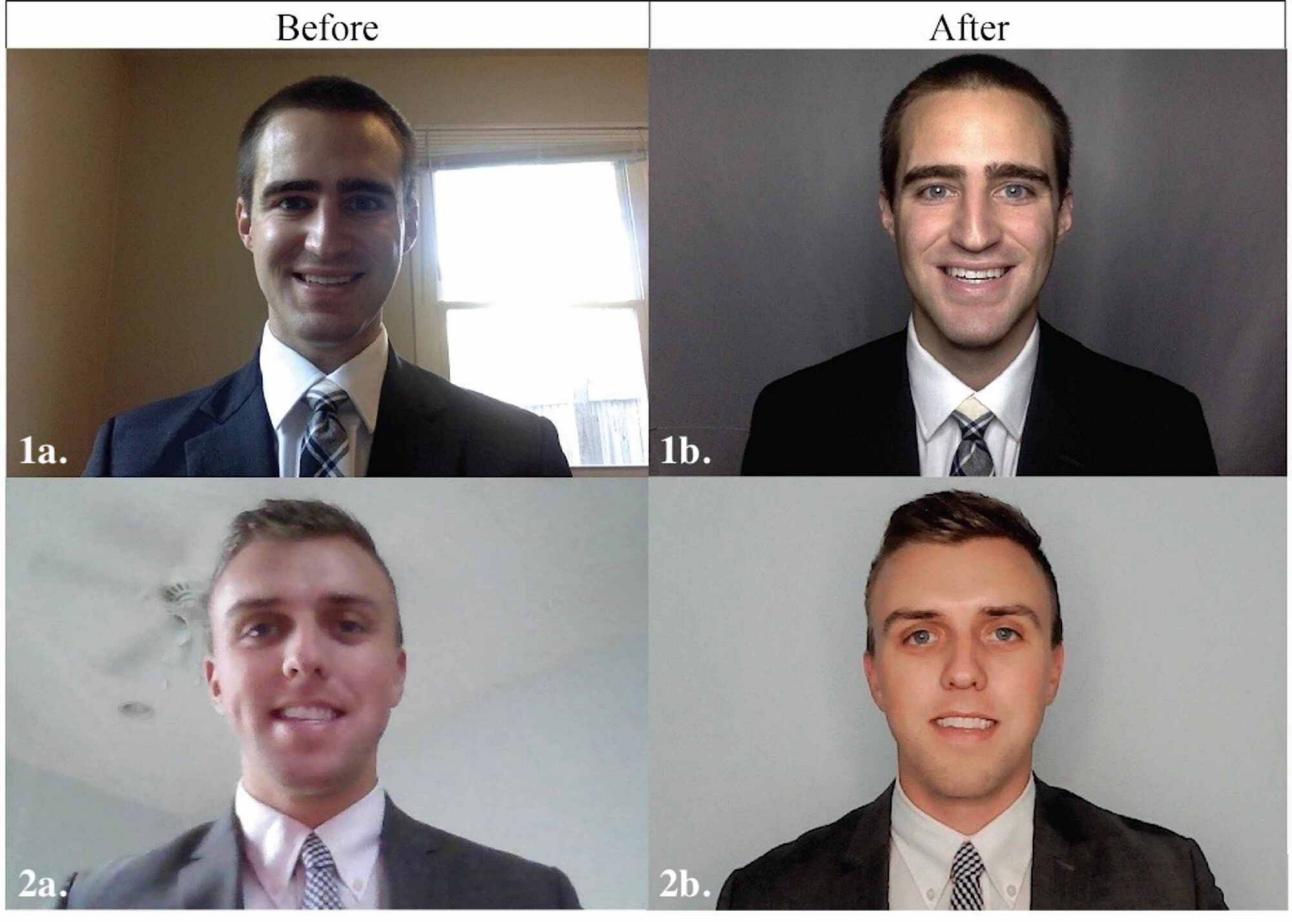
Two examples showing before and after interventions for room environment and audiovisual quality optimization

We sought to create a systematic approach on how to optimize a home interview room, through two examples as shown in Figure 1. 

Our three principles of optimization address the interview room environment and background, audiovisual quality, and virtual interview etiquette. 

## Discussion

Environment and background

The environment of the interview room not only affects the interviewer’s perception of the interviewee, but can also become a distraction to both parties if not controlled. A quiet room, with minimal potential for background noises, is required. Silencing cellphones and computer notifications (such as alerts for incoming emails) will prevent ambient distractions. If in a location with others, consider locking the door and/or placing signage to prevent interruptions. Care of pets or children, who can serve as sources of unpredictability, should be arranged prior to the interview. Consider a non-home interview room if the potential for distracting sounds is high.

We recommend a neutral backdrop for the video call, avoiding backgrounds that include doors or windows. Virtual backgrounds can affect video quality, as well as create a halo effect, in which the outline of the interviewee becomes blurred, and thus should be avoided. A blank wall without pictures or an affordable photography backdrop is optimal options. We recommend a lighter-tone neutral, such as tan or light gray, as shown in Figure 1. 

Audio/visual

Minimum technological requirements to complete a virtual interview include a high definition camera and microphone, and it is imperative to test the audiovisual setup prior to the interview day to identify potential deficiencies. For those without adequate integrated cameras or microphones, many affordable options exist. The camera should be positioned such that it is angled looking slightly down at the interviewee. The slight decline avoids an upward view into the nares of the interviewee and provides a better view of the interviewee’s thorax and extremities to allow for nonverbal cues and gestures, as shown in Figure 1. An easy solution to create this angle is to prop the computer or camera onto textbooks. Backlighting can create an undesirable shadow/silhouette effect. This is mitigated by avoiding light sources emanating from behind the interviewee (e.g. windows), and if needed, using an additional front-facing light. Computer-integrated microphones often sound tinny or capture unwanted background noise. If needed, we recommend an external microphone that is not a headset or headphones, as these can visually distract the interviewer. A lapel-clip microphone, or one with a stand, are both effective options.

A wired internet connection is more stable than a wireless connection and should be utilized via a hardwired ethernet connection directly into the router, if available. If this is impractical, position the router and computer as close to each other as possible or use a signal amplifier to prevent signal degradation. Test the internet speed (via a free online site) at multiple times throughout the day to ensure it can support video conferencing. Importantly, minimize bandwidth issues in advance by coordinating internet use with others. If the specific interview software being utilized is known, create a video session on that platform prior to the interview day. Ensure the software can detect the camera and microphone and that the audio is clear and unmuted. Ideally, recruit a colleague or school administrator/mentor on the video session to verify adequate audiovisual quality.

Etiquette

Virtual interviews should be treated the same as in-personal interviews. From the beginning, it is important to arrive or sign-on early and in professional attire-including the lower half of the body that is not normally seen on the video screen. Prior to the start of the interview, the interviewee should ensure access to water, tissues, pen, and paper to prevent having to leave the video screen for these. If, however, during the course of the interview day, the applicant needs to step away from the computer, we recommend temporarily disabling the camera, as to not confuse interviewers seeing an empty seat. Otherwise, the camera should be kept on at all times with the microphone muted during presentations, but never during interviews. Additionally, the interviewee should remain engaged throughout the entirety of the day, including all presentations in which the applicant is not being interviewed. 

**Figure 2:**
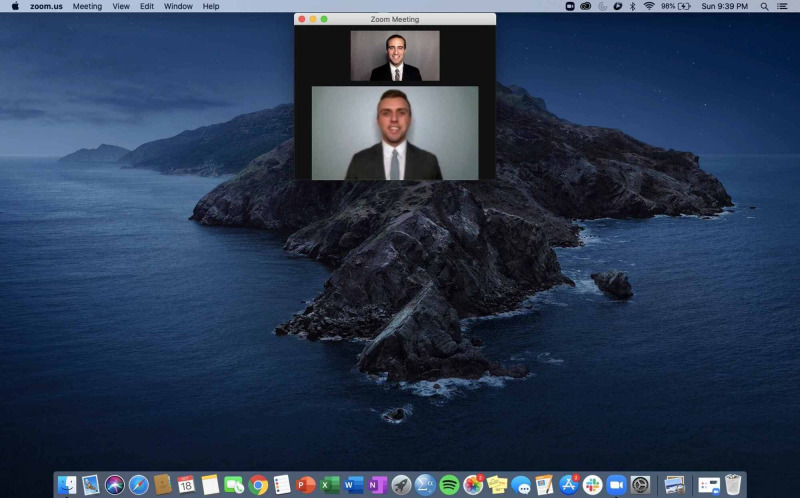
Screen layout to mimic eye contact

Throughout the interview, it is important to smile and make eye contact. While true eye contact is not possible during a virtual interview, our recommendation is to adjust the size of the video window to as small as possible while still being able to adequately see the interviewer, then place this window just below the camera on the computer’s screen as shown in Figure 2. 

This technique will artificially allow the interviewee to be perceived as making eye contact with the interviewer while still being able to assess non-verbal cues from the interviewer. If, during pre-interview testing, this does not adequately mimic eye contact, we recommend looking directly into the camera. Personal notes to reference throughout the interview should be completed on actual paper to avoid the perception that the interviewee is distracted elsewhere on the screen. We recommend showing this notetaking to allow the interviewer to know the interviewee is engaged. 

## Conclusions

Virtual interviews for the 2020-21 residency application cycle provide challenges for both programs and applicants. Through optimization of the interviewee’s environment, audiovisual quality, and overall etiquette, applicants can present the best versions of themselves. While complete optimization of this process may be difficult depending on individual circumstances, the interviewee is encouraged to speak with school administrators regarding available resources and best practices for interviews. 
